# Cardiovascular Effects of Early and Postponed Statin Treatment After Ovariectomy in Prediabetic Rat

**DOI:** 10.33549/physiolres.935584

**Published:** 2026-04-01

**Authors:** Tomas HLINKA, Martina HUTTL, Hana MALINSKA, Irena MARKOVA, Denisa MIKLANKOVA, Hana BARTUSKOVA, Jan PITHA

**Affiliations:** 1 Laboratory for Atherosclerosis Research, Center for Experimental Medicine, Institute for Clinical and Experimental Medicine, Prague, Czech Republic; 2 Internal Department, Faculty Hospital Motol, Prague, Czech Republic; 3 2nd Medical Faculty, Charles University, Prague, Czech Republic; 4 Laboratory for Diabetes Pathology, Center for Experimental Medicine, Institute for Clinical and Experimental Medicine, Prague, Czech Republic; 5 Department of Physiology, Faculty of Science, Charles University, Prague, Czech Republic; 6 Department of Cardiology, Institute for Clinical and Experimental Medicine, Prague, Czech Republic

**Keywords:** Statins, Cardiovascular system, Ovariectomy, Insulin resistance, Rats

## Abstract

We investigated the effects of early and postponed statin therapy after ovariectomy on the cardiovascular system in an experimental model of insulin resistance (hereditary hypertriglyceridemic rats). Thirty hereditary hypertriglyceridemic rats were ovariectomized at 9 weeks of age. Two groups received atorvastatin (5 mg/kg) from the 2^nd^ (early statin) or 14^th^ (postponed statin) week after ovariectomy; the control group received no therapy. Arterial strain was measured by duplex ultrasound at week 21 post ovariectomy. The experiment was terminated at week 22, when the expression of vascular genes was measured together with metabolic and inflammatory parameters associated with the cardiovascular system. Atorvastatin treatment had no positive effect on arterial strain and caused mixed metabolic changes including increased content of triglycerides in striated muscles, and decreased serum levels of monocyte chemoattractant protein-1, fasting glycemia, serum triglycerides and non-esterified fatty acids. The postponed statin group exhibited significantly higher endothelial nitric oxide synthase (eNOS) expression in the myocardium compared to the early statin group, whereas aortic expression was lower in both groups and serum interleukin 6 was moderately decreased. Neither early nor postponed statin therapy improved arterial strain. Early/prolonged atorvastatin treatment was associated with decreased aortic eNOS expression and increased muscle triglyceride content. Postponed/shorter treatment increased myocardial eNOS expression but decreased connexin-43. These findings suggest that in a metabolically impaired state, the timing and duration of statin therapy can result in complex and opposing organ-specific effects.

## Introduction

The specific factors involved in the development of cardiovascular disease (CVD) in women associated with transition to menopause are intensively studied. Women develop CVD 10 years later than men do, but there is a sharp increase in CVD after menopause [1]. The main cause is supposed to be a rapid decrease in estrogen levels during the menopausal transition (MT) but the exact role of sex hormones including mechanisms of their impact on the cardiovascular system is incompletely understood [1,2]. Nevertheless, sex hormone concentrations and their fluctuations within physiological levels seem to be important factors for vascular health both in men and women. Because hormone replacement therapy (HT) after menopause failed to protect women from CVD in two large studies [3,4], it is supposed that appropriate timing of the initiation of estradiol-based HT in the primary prevention of CVD is of the essence and this phenomenon is intensively investigated [5]. In general, in more advanced stages of atherosclerosis, HT could have a detrimental effect on the vascular system [6]. Nevertheless, still no data is available for timing of the treatment of main traditional cardiovascular risk factors including dyslipidemia. In this respect, our observational study in pre-, peri- and postmenopausal women showed that smoking habits have the most detrimental effect if present during perimenopause [7].

To simulate the clinical status of women at increased cardiovascular risk approaching menopause, we used ovariectomized hereditary hypertriglyceridemic (HHTg) rats as a model for metabolic syndrome and insulin resistance (8). In this model we tested the hypothesis that early initiation of statin therapy shortly after ovariectomy might prevent negative vascular and metabolic changes compared to postponed therapy and even in the presence of metabolic syndrome the benefits of statin therapy might prevail.

## Materials and Methods

### Animals and diet

The Prague hereditary hypertriglyceridemic (HHTg) rat was chosen as a prediabetes model because it exhibits genetically determined hypertriglyceridemia, insulin resistance in peripheral tissues, impaired glucose tolerance and hepatic steatosis but is not associated with obesity or fasting hyperglycemia [9]. In addition, ovariectomized rats are already established as an experi-mental model of menopause, displaying metabolic and functional changes related to ovarian cycle loss [10–12].

Thirty female HHTg rats (experimental units) bred by the Institute for Clinical and Experimental Medicine, Prague, Czech Republic and approved by the research ethics committee (Protocol Number 24/2021) were included in this study. After weaning, animals were randomly divided into breeding cages. All young animals were placed in a large container and then randomly allocated to their new experimental families (breeding cages), where they remained until the end of the experiment. Each animal was marked with a tail strip and had an identification card (Protocol of the Experimental Project).

All experimental procedures were approved by the Experimental Project Committee, which also considered the minimization of potential confounders. Experiments were performed by an experienced, trained team. Animals were randomized, and samples for biochemical and histological analysis were evaluated blindly. Due to logistical constraints, it was not possible to control all other potential confounding factors.

There were no a priori criteria for including/excluding animals, units or data results in our study, and no animals were excluded. During allocation, conduct of the experiment, outcome assessment (including ultrasonography and biochemical analyses), and data analysis, all personnel were blinded to the group assignments.

The animals were kept under conditions of controlled temperature (22 °C), humidity, and lighting (12/12 h light/dark cycle) with free access to a standard chow diet and drinking water.

At 9 weeks of age, all rats were weighed and anesthetized using ketamine (70 mg/kg) and xylazine (10 mg/kg) administered intraperitoneally and then bilaterally ovariectomized via midline incision. The animals were saturated with oxygen, and postprocedural analgesia was provided using meloxicam (1 mg/kg). After that, they were randomly divided into three groups (n=10). Two groups were treated with atorvastatin (5 mg/kg) at the 2nd (early statin – ES) or 14th (postponed statin – PS) week after ovariectomy and one group was not exposed to treatment and served as a control.

The drug was mixed with the standard chow diet (Altromin, Maintenance diet for rats and mice, Germany).

The sample size for each experimental group was determined based on similar experiments conducted in our department. The initial number of animals in each group was sufficient to achieve statistical significance and adhered to the 3R principles of Replacement, Reduction, and Refinement.

During the 21st week after ovariectomy, an examination by duplex ultrasound was performed in all animals under study. In the following week, all rats were sacrificed by decapitation in the postprandial state. Serum and tissue samples were then collected and stored at −80 °C for further analyses.

**Fig. 1:**
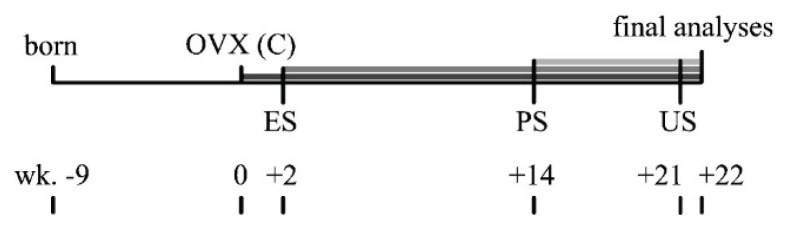
Timeline of the study. Weeks (wk.) are shown relative to the week of ovariectomy. OVX – week 0. C – establishing the control group, ES – starting statin treatment in the early treated group, PS – starting statin treatment in the postponed group, US – ultrasonography measurements of all the groups.

The timeline of the study is shown in Fig. 1.

### Ultrasound evaluation

Ultrasound measurements were performed using the US imaging system (Vevo 2100, FUJIFILM, VisualSonics Inc., Toronto, Canada). Isoflurane anesthesia was given in an induction chamber and maintained with a nose cone (1.5–2 % isoflurane in 0.4–0.8 l/m oxygen). The animals were then shaved and positioned on a temperature-controlled plate. ECG, breathing frequency, and board temperature were continuously monitored throughout the examination. An ultrasonographic probe (Vevo MS250S for the carotid artery and MS550D for the abdominal aorta) was applied to either the epigastric region or the neck. Using B-mode and M-mode after optimization, three sequential recordings were obtained for each vessel. VevoLab software (FUJIFILM, VisualSonics Inc., Toronto, Canada) was used to measure vessel diameter. The data were then exported using VevoLab software and analyzed using Microsoft Excel for Microsoft 365. Strain was expressed as the percentage change in vascular diameter and calculated for each heart cycle as (SD-DD)/DD. SD is the systolic diameter, DD is the diastolic diameter.

### Biochemical analysis in serum

Serum levels of glucose, triglycerides (TG), nonesterified fatty acids (NEFAs), total and high-density lipoprotein (HDL) cholesterol were measured using commercially available kits (Erba Lachema, Brno, Czech Republic; Roche Diagnostics, Germany). For serum creatinine measurements, an enzymatic spectro-photometric method was used (a kit from Roche Diagnostics, Germany). Serum monocyte chemoattractant protein-1 (MCP-1), IL-6 and nitric oxide synthase (NOS) concentrations were determined using rat ELISA kits (Invitrogen, Thermo Fisher Scientific, Waltham, MA, USA; MyBiosource, San Diego, CA, USA).

Serum 17β-estradiol (kit number: DLS 4800), 17β-hydroxyprogesterone (kit number: IM 1452) and testosterone (kit number: IM1087) were analyzed using rat RIA kits (Immunotech, Prague, Czech Republic).

### Tissue triglyceride and cholesterol measurements

For TG in muscle, tissue samples were powdered under liquid N_2_ and extracted in chloroform/methanol. Then, a solution of 2 % potassium dihydrogen phosphate was added, the mixture was centrifuged, and the organic phase was removed and evaporated under N_2_. The resulting pellet was dissolved in isopropyl alcohol, and the TG content was measured by an enzymatic assay (Erba-Lachema, Brno, Czech Republic).

### Gene expression assays

Total RNA was isolated from abdominal aorta and myocardial left ventricle tissue using RNA Blue (Top-Bio, Vestec, Czech Republic). Reverse transcription and quantitative real-time PCR analysis were performed using the TaqMan RNA-to-C_T_ 1-Step Kit and TaqMan Gene Expression Assay (Applied Biosystems, Waltham, MA, USA). Relative expression levels of eNOS (assay ID: Rn02132634_s1), *Connexin37 (Cx-37)* (assay ID: Rn00572193_s1), IL-6 (assay ID: Rn01410330_m1), MCP-1 (assay ID: Rn00580555_m1) and Connexin43 (Cx-43) (assay ID: Rn01433957_m1) were determined after normalization against the Hprt (Hypoxanthine-guanine phosphoribosyltransferase) (assay ID: Rn01527840_m1) gene as an internal reference and calculated using the 2^−^^ΔΔ^^Ct^ method, with results run in triplicate.

### Statistics

The data are presented as the mean ± standard deviation. To detect differences between the groups under study, one-way ANOVA was used. Prior to conducting the ANOVA, we assessed the assumptions of normality, homogeneity of variance, and independence. Tukey's post hoc test was used for pairwise comparisons when appropriate. Statistical significance was defined as p < 0.05. All the statistical analyses were performed using Microsoft Excel for Microsoft 365, R Studio and GraphPad Prism 9.0.0.

## Results

We did not observe any effect of atorvastatin treatment, including its timing, on body weight (Table 1).

**Fig. 2:**
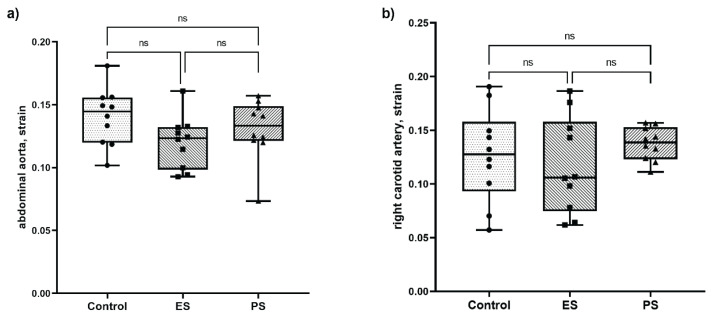
Strain of the (**a**) abdominal aorta and (**b**) right common carotid artery. Strain (the relative change in the vessel diameter from diastole to systole) was calculated as the difference between the systolic and diastolic diameters of the vessel, divided by the diastolic diameter. Measurements were acquired using ultrasound. The significance level was determined through the use of one-way ANOVA and Tukey's honest significant difference test. The level of significance is indicated by asterisks (* p<0.05, ** p<0.01, *** p <0.001), with lower p-values corresponding to greater significance. Control – control group, ES – early statin treated group, PS – postponed statin treated group

**Fig. 3:**
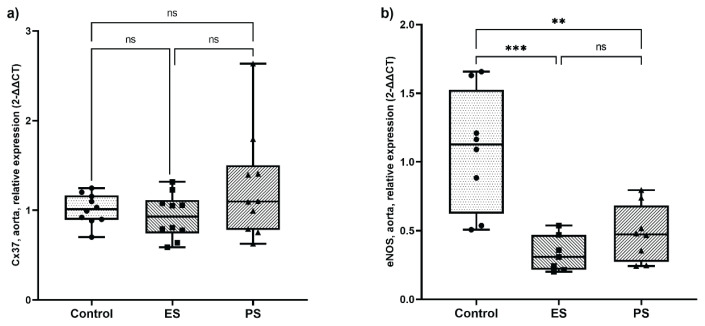
Relative expression of the **a**) Connexin 37 (Cx37) and **b**) eNOS gene in the aorta. Relative expression of proteins was evaluated by qPCR and is expressed as 2–ΔΔCt. The significance level was determined through the use of one-way ANOVA and Tukey's honest significant difference test. The level of significance is indicated by asterisks (* p<0.05, ** p<0.01, *** p <0.001), with lower p-values corresponding to greater significance. Control – control group, ES – early statin treated group, PS – postponed statin treated group.

The strain of the abdominal aorta and of the right carotid artery did not differ between the ES and PS groups (Fig. 2). Both treated groups had similar values of Cx-37 expression as the control group, but eNOS gene expression in both treated groups was significantly lower than that in the control group (Fig. 3).

**Fig. 4:**
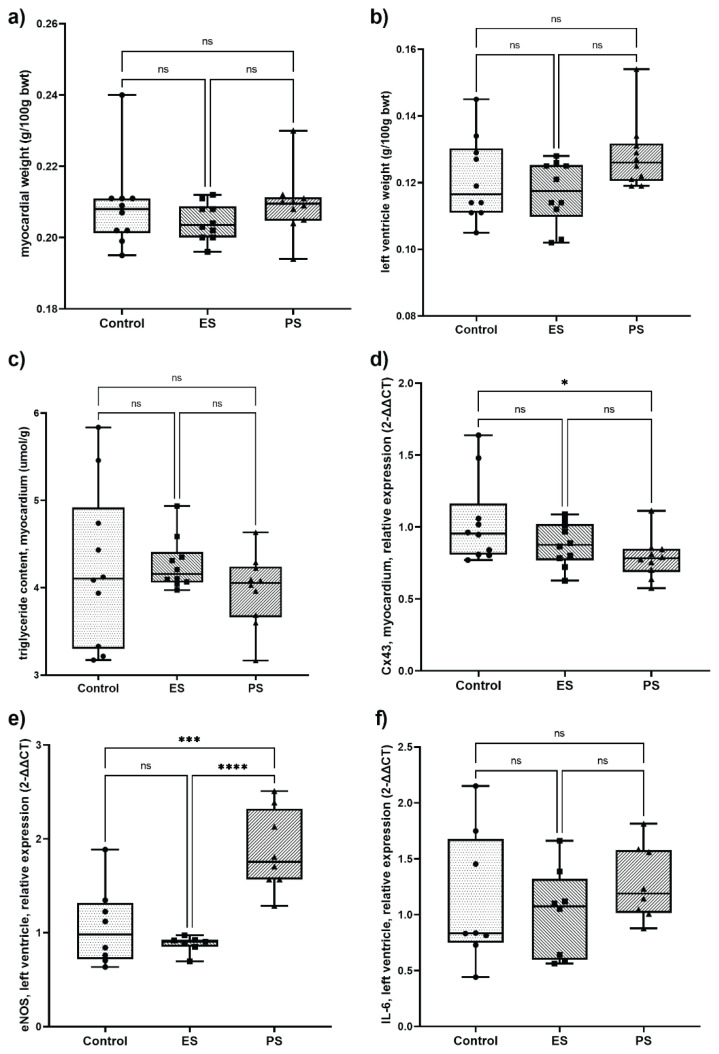
Myocardial parameters: **a**) relative myocardial weight, **b**) relative weight of the left ventricle, **c**) triglyceride content, and relative expression of **d**) connexin 43 (Cx43) in the myocardium, **e**) eNOS gene in the left ventricle and **f**) interleukin 6 (IL-6) in the myocardium. Relative expression of proteins was evaluated by qPCR and is expressed as 2–ΔΔCt. The significance level was determined through the use of one-way ANOVA and Tukey's honest significant difference test. The level of significance is indicated by asterisks (* p<0.05, ** p<0.01, *** p <0.001), with lower p-values corresponding to greater significance. eNOS - endothelial nitric oxide synthase 3, bwt – body weight, Control – control group, ES – early statin treated group, PS – postponed statin treated group

The weight of the myocardium, including the left ventricle, and TG content in the myocardium did not differ between the ES and PS groups (Fig. 4a–c). No differences in Cx-43 expression were detected between the ES and PS groups; however, there was a significant decrease in the PS group compared to the control group (Fig. 4d). The expression of the eNOS gene in the left ventricle was significantly lower in the ES group than in the PS group; it was similar between the ES and control groups, but significantly higher in the PS group compared to the control group (Fig. 4e). No significant differences were observed in the relative expression of IL-6 in the left ventricle among the study groups (Fig. 4f).

The concentration of TG in striated muscle was significantly greater in the ES than in the PS group. In addition, both statin-treated groups had higher values than the control group.

Regarding blood lipid parameters, the concentrations of total cholesterol were significantly higher in the ES group than in the PS group. The values in the PS group were similar to those in the control group, and the values in the ES group were greater than those in the control group.

The serum TG and NEFA levels did not differ between the ES and PS groups, but they were significantly lower in the statin-treated groups than in the control group. No between-group differences were found for HDL cholesterol. Fasting glycemia was lower in the ES group than in the PS group, and the PS group had similar values to the control group. The serum concentration of IL-6 did not differ between the groups. However, the serum concentration of MCP-1 was significantly lower in both treatment groups than in the control group. The ES and PS groups showed similar values. Regarding other parameters under study, no between-group differences were observed (serum creatinine, estradiol, progesterone, and testosterone). All the results are shown in Table 1 and 2.

The data are presented as the mean ± SD. One-way ANOVA was utilized to examine the differences between groups and a post hoc test (Tukey HSD) was used when appropriate. Statistical significance was defined as p < 0.05. Tukey HSD - Tukey's honest significance test, TG – triglyceride, NEFA – non-esterified fatty acid, MCP-1 - monocyte chemoattractant protein-1, IL-6 – interleukin 6, NOS - nitric oxide synthase, HDL -high-density lipoprotein, Control – control group, ES – early statin treated group, PS – postponed statin treated group.

## Discussion

We observed no significant effect of early statin treatment after ovariectomy on vascular function expressed as the strain of the abdominal aorta or right carotid artery in an experimental rat model of prediabetes compared to postponed statin treatment.

In addition, aortic eNOS expression did not differ between the two statin-treated groups, and it was significantly lower in these groups than in the control group.

In the myocardium the effects of therapy were more complex. Most parameters, including weight of the left ventricle and TG content, remained unchanged by statin treatment but we observed two significant changes in gene expression in the postponed statin (PS) group.

First, on one hand, the shorter statin treatment duration in the PS group led to a favorable increase in myocardial eNOS expression compared to both the control and ES groups. Second, on the other hand, the PS group exhibited a significant decrease in the expression of connexin 43, a gap junction protein essential for coordinating cardiomyocyte contraction and electrical signaling. A reduction in connexin 43 has been linked to an increased risk of arrhythmias and impaired cardiac function. Therefore, the seemingly beneficial effect on eNOS in the PS group was accompanied by a potentially detrimental change in a key protein for cardiac conductivity. This might reflect complex and different effects of statin therapy at the myocardial level in this specific experimental model. Based on these findings, the described increase of myocardial eNOS must be interpreted with caution.

The limited efficacy of statin treatment in this given experimental model might be attributed to the rapid onset of pathological changes following ovariectomy, suggesting that even the early treatment might be initiated too late.

These findings were rather surprising because statins are supposed to have robust positive effects on cardiovascular function, including increasing vascular eNOS, and nitric oxide (NO) bioavailability and suppressing proinflammatory factors such as tumor necrosis factor α (TNF-α) and interleukins [13], which were not affected by statin treatment in our study (see below).

Another surprising finding was the accumulation of triglycerides (TG) in striated muscle, a marker of ectopic fat deposition often associated with insulin resistance. Both treated groups showed a significant increase in muscle TG content compared to the control group and the effect was more pronounced with the early treated group. This suggests that atorvastatin itself might promote ectopic lipid storage in muscle, a finding consistent with other studies linking statin therapy to altered lipid metabolism in diabetic states.

Ovariectomy is known to have a negative effect on glucose metabolism and also causes skeletal muscle contractile dysfunction, potentially via impaired skeletal muscle insulin sensitivity, increased oxidative stress, and mitochondrial dysfunction in skeletal muscle and these known effects form the physiological background for our experimental model [14–16].

Additionally, other metabolic changes in this experimental model, potentially influenced by statin administration, were observed, including reduced peripheral insulin sensitivity, impaired glucose metabolism and reduced myocyte mitochondrial function [17,18]. This strain is also known to have reduced expression of the adipocyte insulin-responsive glucose transporter type 4 (GLUT4) which plays a major role in the pathophysiology of type 2 diabetes mellitus [19–22]. The available literature has also linked the aforementioned metabolic disturbances to the accumulation of excess lipids in organs such as the liver, skeletal muscle, pancreas, and heart, also referred to as ectopic fat deposition [23].

One of the potential explanations for our findings is the diabetogenic effect of statins as they could further contribute to impaired insulin metabolism. Therefore, their prolonged application in already preexisting insulin resistance may attenuate their otherwise positive vascular effects and potentiate untoward changes in carbohydrate metabolism [24]. This is supported by data showing that lower cellular cholesterol content could impair insulin secretion by disrupting voltage-gated calcium channel function in pancreatic beta cells [17,25], thereby reducing the fusion of insulin granules with the cell membrane for subsequent export and indicating at least an indirect effect of statins. The intracellular cholesterol content in this particular study was not measured, but this possibility cannot be excluded.

The evidence providing partial and indirect support for these considerations stems from several available experimental studies. Singh *et al.* [26] observed in mice that atorvastatin selectively impaired mitochondrial function in glycolytic muscle and Bouitbir *et al.* [27,28] observed in their rat experiment that treatment with atorvastatin was associated with mitochondrial oxidative stress and apoptosis and contributed to myopathy in glycolytic muscles, highlighting their heightened sensitivity compared to that of oxidative muscles. A similar effect of statins on skeletal muscles was described in a mouse model of type-1 diabetes by Rebalka *et al.* [29]. They demonstrated a significant increase in skeletal muscle ectopic lipid droplets and significant decreases in lipid transporter content within the skeletal muscle of statin-treated mice (Streptozotocin-induced diabetic mice – STZ-induced mice). Moreover, none of these findings were detected in statin-treated, nondiabetic (wild-type) mice. Using ZDF mice Bonen *et al.* [30] also proposed that, as is the case with GLUT4, FAT/CD36 cycling is impaired in insulin-resistant skeletal muscle. FAT/CD36 is retained on the cell surface, which results in an increased rate of LCFA transport [31].

From a human perspective, Bonen *et al.* [31] showed that in patients with type 2 diabetes skeletal muscle TG concentrations were increased two- to threefold, which might be due to markedly increased rates of long-chain fatty acid (LCFA) esterification and that there were positive associations between skeletal muscle TG deposits and rates of LCFA transport into giant vesicles and FAT/CD36 protein expression at the plasma membrane. In addition, Phillips *et al.* [32] showed abnormally elevated lipid stores, ragged red fibers and fibers with decreased staining for cytochrome c activity as a marker of impaired mitochondrial activity in muscle biopsies from three patients with symptoms of myopathy [25].

The further potentiation of insulin resistance by ovariectomy in our study is supported by the increased accumulation of TG in striated muscles, the known deficiency of the GLUT4 transporter in HHTg rats was already studied in this model [22] in which in another study, atorvastatin-treated animals showed a significant increase in muscle TG concentration even without ovariectomy [33]. In our previous study, there was no increase in muscle TG content after ovariectomy with estradiol substitution and without statin treatment [10].

Based on these data, we hypothesize that the absence of an unequivocal favorable effect of atorvastatin on the cardiovascular system might also be explained by the already present negative metabolic status of our model, further potentiated by ovariectomy, rendering it more resistant to statin treatment.

We also analyzed the effect of statins on two proinflammatory cytokines, IL-6 and MCP-1 [34,35]. Both statin-treated groups showed moderate increases in circulating IL-6 concentrations and we did not find any significant differences in the expression of IL-6 in the myocardium. In human studies, the concentration of IL-6 increases after menopause, most likely due to estrogen deprivation [36]. In our previous study in HHTg rats, we also detected increased IL-6 levels immediately after ovariectomy in the same model [10]. Therefore, we propose that during the early stages of vascular impairment, IL-6 may play an ambiguous role; its upregulation could also represent compensatory mechanisms rather than solely deleterious effects on the vascular system [37]. This possibility should be considered especially given the fluctuating sex hormone levels during menopause transition [38]. While statin administration is generally accepted to exert positive pleiotropic actions beyond their effects on lipid lowering (for example decrease the expression and function of proatherogenic and proinflammatory cytokines IL-6, MCP-1, TNF-α), after menopause and in the milieu of metabolic impairment, these effects might be attenuated as was already reviewed [35,39].

In contrast to IL-6, we observed significantly lower concentrations of MCP-1 in both statin-treated groups than in the control group but, at the same time, we detected no difference between the ES and PS groups (Table 2). This effect can be explained by effects on the angiotensin II receptor type 1 (AT1R)/Geranylgeranyl pyrophosphate (GGPP)/Rho pathway and on peroxisome proliferator-activated receptors (PPARs) and chemokine receptors [34,35].

Regarding interconnection between both cytokines under study, previous studies have suggested that MCP-1 and IL-6 function independently, but recent studies have shown their codependence [40–43] and potential for mutual stimulation, which is dependent on the surrounding environment [40]. We, therefore, hypothesize that MCP-1 might be a more reliable indicator of statin's anti-inflammatory effect than IL-6.

## Limitations of the study

Our results are based on a specific rat model of metabolic syndrome and may not be directly translatable to postmenopausal populations. However, HHTg rats were bred specifically to be a non-obese model of hypertriglyceridemia and genetic analysis shows that the traits in the HHTg rats share a genetic basis with similar conditions in humans. In addition, previous research on the HHTg rat model has already demonstrated cardiovascular benefits from statin therapy [44,45]. The study lacks a non-ovariectomized control group, so the direct effects of ovariectomy cannot be safely inferred from this study, but it was described in our previous study in the same model [10].

## Conclusions

In female HHTg rats, early exposure to atorvastatin after ovariectomy did not improve the strain of the abdominal aorta and carotid artery; it did not change eNOS expression in myocardial tissue; furthermore, it decreased eNOS expression in the abdominal aorta and led to an increase of the triglyceride content in striated muscles. In contrast, shorter exposure in postponed atorvastatin treatment after ovariectomy led to increased expression of eNOS in myocardial tissue, but other cardiovascular parameters did not show improvement, therefore, we cannot exclude a chance finding.

These findings suggest that the timing and duration of atorvastatin therapy can lead to divergent and organ-specific effects in this given model of prediabetes and menopause.

## Figures and Tables

**Table 1:** Basal metabolic parameters in serum, and muscle triglycerides

	Control (n=10)	ES: 2nd week (n=10)	PS: 14th week (n=10)
*Body weight (g)*	300 ± 6	285 ± 3	282 ± 9
*TG (mmol/l)*	1.53 ± 0.14	1.06 ± 0.06	1.20 ± 0.09
*Total cholesterol (mmol/l)*	1.62 ± 0.04	1.74 ± 0.02	1.59 ± 0.04
*HDL-cholesterol (mmol/l)*	0.59 ± 0.01	0.63 ± 0.01	0.59 ± 0.02
*NEFA (mmol/l)*	0.75 ± 0.03	0.65 ± 0.04	0.55 ± 0.04
*Serum creatinine (mmol/l)*	28.90 ± 1.20	27.30 ± 0.6	29.10 ± 0.90
*Fasting glucose (mmol/l)*	5.70 ± 0.20	5.20 ± 0.1	5.50 ± 0.10
*Testosterone (ng/ml)*	0.066 ± 0.010	0.084 ± 0.016	0.09 ± 0.008
*17* *β* *-estradiol (pg/ml)*	13.03 ± 0.75	15.07 ± 1.13	13.16 ± 1.32
*17* *β* *-hydroxyprogesterone (ng/ml)*	0.36 ± 0.042	0.32 ± 0.04	0.39 ± 0.05
*MCP-1 (ng/ml)*	5.39 ± 0.32	4.30 ± 0.20	4.19 ± 0.18
*IL-6 (pg/ml)*	61.60 ± 4.90	87.40 ± 7.00	68.90 ± 10.60
*NOS (ng/ml)*	0.73 ± 0.20	0.77 ± 0.18	1.02 ± 0.36
*TG muscle (μmol/g)*	1.85 ± 0.42	3.52 ± 0.74	2.62 ± 0.61

**Table 2:** Basal metabolic parameters in serum, and muscle triglycerides: statistics

	p (ANOVA)	Tukey HSD ES vs. PS (p)	Tukey HSD ES vs. Control (p)	Tukey HSD PS vs. Control (p)
*Body weight (g)*	n.s.	-	-	-
*TG (mmol/l)*	< 0.01	n.s.	< 0.01	n.s.
*Total cholesterol (mmol/l)*	< 0.05	< 0.05	< 0.05	n.s.
*HDL-cholesterol (mmol/l)*	n.s.	-	-	-
*NEFA (mmol/l)*	< 0.01	n.s.	n.s.	< 0.01
*Serum creatinine (mmol/l)*	n.s.	-	-	-
*Fasting glucose (mmol/l)*	< 0.001	< 0.05	< 0.001	n.s.
*Testosterone (ng/ml)*	n.s.	-	-	-
*17* *β* *-estradiol (pg/ml)*	n.s.	-	-	-
*17* *β* *-hydroxyprogesterone (ng/ml)*	n.s.	-	-	-
*MCP-1 (ng/ml)*	< 0.01	n.s.	< 0.01	< 0.01
*IL-6 (pg/ml)*	n.s.	-	-	-
*NOS (ng/ml)*	< 0.05	n.s.	n.s.	< 0.05
*TG muscle (μmol/g)*	< 0.0001	<0.01	< 0.0001	< 0.05
